# Toward the Use of Centrifugation for Point-of-Care Plasma Separation: Evaluation of a Compact Battery-Powered Centrifuge

**DOI:** 10.3390/diagnostics16193088

**Published:** 2026-09-24

**Authors:** Adam S. Ptolemy, Alexander L. Kolb, Ayse Mehmke, Sami Rifai, Nira R. Pollock

**Affiliations:** Department of Laboratory Medicine, Boston Children’s Hospital, Boston, MA 02115, USA

**Keywords:** point-of-care testing, sample collection, blood testing, plasma, centrifugation, pre-analytics, Microtainer

## Abstract

**Background/Objectives:** Despite advances in point-of-care (POC) blood test development, the lack of a simple, low-cost approach to the separation of capillary blood to generate liquid plasma remains a barrier to POC measurement of many analytes. The battery-operated, internally balanced Labcorp TrueSpin^®^ centrifuge offers a potential POC solution for blood collected into a BDMicrotainer^®^. We compared the quality of plasma generated by TrueSpin centrifugation versus conventional centrifugation. **Methods:** Venipuncture was performed on 10 volunteers. After collection of blood into Sarstedt Monovettes^®^ (Lithium Heparin with gel (LiHep/Gel), EDTA), blood from the same tubing was collected into three Microtainers (LiHep/gel, LiHep without gel (LiHep), EDTA). Microtainer and Monovette samples received TrueSpin and conventional centrifugation, respectively. Hemolysis index and plasma amino acids (*n* = 24) were measured for all Monovette and Microtainer plasma samples. For 3 additional volunteers, capillary blood was collected from the arm into LiHep/Gel and EDTA Microtainers using the Labcorp Comfort Draw^TM^ device with a gentleheel^®^ lancet and centrifuged (TrueSpin) to generate plasma. **Results:** TrueSpin centrifugation took approximately 10 min; batteries supported 8–11 runs. Microtainer plasma H-indices (including blood from Comfort Draw) were ≤10. For all 24 amino acids combined, measurements for TrueSpin Microtainer samples had excellent Pearson’s correlation (r > 0.99) with paired conventional Monovette plasma. **Conclusions:** Plasma quality after TrueSpin centrifugation of Microtainer blood samples is comparable to plasma quality after conventional Monovette centrifugation, including for capillary blood collected using the Comfort Draw. The TrueSpin was reliable and easy to use. The TrueSpin centrifuge has potential future use for POC plasma separation.

## 1. Introduction

Recent advances in the use of large-volume capillary samples for testing of blood at the point-of-care (POC) have catalyzed interest in the development of tools to produce and collect such samples [1]. The granting of marketing authorization by the Food and Drug Administration (FDA) to the Xpert HCV test, which utilizes 100 µL of fingerstick blood collected into an EDTA Microtainer^®^ (Becton, Dickinson, and Company; Franklin Lakes, NJ, USA) with a 250–500 µL volume range [2], provides a new precedent for use of Microtainer blood samples for POC testing. Multiple FDA-cleared tools now exist for production of large volume capillary samples, including tools designed for yielding blood from the upper arm rather than the finger, and these tools can be combined with EDTA- or heparin-coated capillary collection containers for blood capture [1]. However, separation of capillary whole blood into liquid plasma to be used for testing at the POC remains a major barrier to the development and deployment of POC tests utilizing blood.

The Labcorp TrueSpin^®^ centrifuge (Labcorp; Burlington, NC, USA) is a class I, FDA-registered, battery-operated, benchtop device capable of separating a Microtainer blood sample into serum or plasma in approximately 10 min [3]. Notably, the centrifuge has a pre-set spin rate and does not need to be balanced by an operator, raising the possibility of use by an untrained individual in a CLIA-waived setting. The TrueSpin has been demonstrated to produce high-quality serum from both venous and capillary whole blood samples collected into Microtainer serum separator tubes (SST) [3]. Another small study also demonstrated that lay users were able to self-collect blood (using a Tasso+ device), use the TrueSpin centrifuge to separate serum, and even ship the serum sample to LabCorp using overnight shipping [4], opening the door to use of such a workflow for waived testing.

Notably, the ability of the TrueSpin to specifically generate plasma has not yet been assessed; nor has the quality of such plasma been evaluated. Both EDTA and Lithium Heparin (LiHep) plasma have utility for POC testing, as EDTA and LiHep are the primary anticoagulants used to generate plasma for clinical testing for a broad range of analytes and use cases. In particular, the ability to easily generate EDTA plasma from Microtainer samples at the POC would substantially advance POC molecular testing, including for many important infectious diseases. The use of tubes with a gel barrier separating plasma from cellular components would also substantially facilitate POC use by simplifying plasma removal after centrifugation. In this study, we evaluated the quality of plasma produced by TrueSpin centrifugation of venipuncture whole blood collected directly into the three types of anticoagulant-coated Microtainers that are available commercially [LiHep with gel (LiHep/Gel), LiHep without gel (LiHep), and EDTA] and compared the plasma to that produced by conventional centrifugation of venipuncture whole blood collected into Sarstedt Monovette^®^ (Nuembrecht, Germany) evacuated collection tubes (LiHep/Gel or EDTA). To assess plasma quality in this feasibility study, we evaluated hemolysis and measured 24 different amino acids (AA) in plasma using a laboratory-developed assay [5] in use for clinical testing at our hospital. Looking towards a future POC workflow, we also performed a proof of principle evaluation using the Labcorp Comfort Draw^TM^ (Labcorp; Burlington, NC, USA) device to collect capillary blood from the arm into LiHep/Gel and EDTA Microtainers prior to TrueSpin centrifugation.

## 2. Materials and Methods

Venipuncture was performed on 10 volunteers using a butterfly needle and attached tubing. Volunteers were laboratory staff participating in a routine clinical laboratory test validation project and no specific inclusion criteria were utilized. After collection of blood into two Sarstedt Monovettes (LiHep/Gel, then EDTA), additional blood was collected from the same venipuncture tubing into three Microtainers [LiHep/Gel (BD 365985), LiHep (BD 365965), and EDTA (BD 365974)]. The order of collection of the Microtainer samples was rotated for each collection. Microtainer samples were filled and inverted per their respective instructions for use. Given that dripping of blood from the tubing into the Microtainer was quite fast, the sample volume collected was occasionally in excess of the maximum volume for the Microtainer; in this case, after inversion, excess volume was removed by pipette to reach the maximum volume line. All Microtainer samples underwent TrueSpin centrifugation, followed by plasma removal and measurement with a calibrated pipettor; Monovette samples underwent conventional centrifugation (2000× *g* for 10 min) to separate plasma, which was also removed with a calibrated pipettor.

The hemolysis index (H-index) was measured using an aliquot of plasma isolated from each Monovette and Microtainer blood sample within 1 h of collection using the sample index assay (Sample Index Gen 2, SI2) from Roche Diagnostics (Indianapolis, IN, USA) on a Cobas^®^ c503 Pro automated clinical chemistry analyzer. The H-index measuring range for this assay is 5–1000, with 5 representing the lowest measurable analyte level that can be distinguished from 0.

Plasma AA were quantified using an established ultra-performance liquid chromatography with UV detection (UPLC-UV) assay, which has been previously described [5]. Briefly, an Acquity™ UPLC with an integrated UV detector and a commercially available MassTrak™ AAA Solutions kit (Waters Corporation, Milford, MA, USA) were used throughout the study. Pre-column AA derivatization with a 6-aminoquinolyl-N-hydroxysuccinimide carbamate tag (AccQTag^®^) was performed according to manufacturer instructions. Prepared plasma specimens were injected into a MassTrak™ AAA column (2.1 × 150 mm, 1.7 µm, Waters Corporation), with the derivatized products separated using a gradient and detected by an in-line UV detector (λ_abs_: 260 nm). The following plasma amino acids (*n* = 24) were evaluated with this LC-UV protocol: alanine (Ala), glycine (Gly), isoleucine (Ile), leucine (Leu), valine (Val), serine (Ser), threonine (Thr), arginine (Arg), histidine (His), hydroxyproline (Hyp), lysine (Lys), asparagine (Asn), glutamine (Gln), aspartic acid (Asp), glutamic acid (Glu), phenylalanine (Phe), tryptophan (Trp), tyrosine (Tyr), citrulline (Cit), cystine, ornithine (Orn), taurine (Tau), methionine (Met), and proline (Pro). External MassCheck^®^ Amino Acid Analysis Plasma Controls (AAA QC; Chromsystems, Munich, Germany) were included in every analytical run to assess the accuracy of the LC–UV batch testing procedure, which was also used for routine patient testing at our institution. The inter-day precision (%CV) of the matrix-matched Level I AAA QC, *n* = 141 measurements collected from January to June 2026 and encompassing the study testing dates, ranged from 2.3% for Phe, Ile, Leu and Trp to 6.4% for Tau. Over the same period, the achieved %CV for the Level III AAA QC ranged from 2.7% for Val to 5.9% for Tau. The mean measured concentrations of all 24 amino acids in both the Level I and Level II AAA QC materials during this period were within the manufacturer’s respective acceptable ranges. Aliquots of all paired plasma samples derived from each individual were stored frozen and then tested with the LC-UV plasma amino acid assay.

Operational characteristics assessed for the TrueSpin centrifugation process included centrifugation time, centrifuge error, movement of the centrifuge on the bench during rotation (with and without a prototype base provided partway through the study), visual separation of plasma, and visual assessment of hemolysis.

Comfort Draw capillary blood production and collection into EDTA and LiHep/Gel Microtainers was performed using the manufacturer’s instructions for use (Labcorp, 2000000045), including use of the gentleheel lancet (Cardinal Health, TGH10X50; Dublin, OH, USA). Arm warming time using heat packs (Medzah, Non-Woven Instant Heat Pack; Glastonbury, CT, USA) ranged from 3–5 min. Microtainer centrifugation, plasma removal, and H-index/amino acid testing were performed as described previously; all TrueSpin centrifugation was performed using the prototype base.

We plotted paired values (e.g., alanine concentration from EDTA Sarstedt Monovette plasma versus alanine concentration from EDTA Microtainer plasma) for all (*n* = 24) amino acids tested in each sample on the same plot, allowing visualization of correlation over the entire study population in one plot. The distribution of the pooled amino acid concentrations was assessed descriptively rather than by formal normality testing. The pooled data exhibited right-skewness, reflecting the broad range of physiological concentrations represented by the 24 plasma amino acids. All method agreements in aggregate were evaluated using Passing-Bablok regression, with the 95% confidence interval (CI) of the slope and intercept calculated, and Pearson’s correlation. Bland-Altman plots also quantified these agreements, with the mean concentration difference and limits of agreement (LoA, ±1.96 standard deviations (SD)) calculated. Pearson’s correlation was used because it retains the quantitative magnitude of plasma amino acid concentrations while evaluating linear correspondence between methods. Statistical calculations were performed using R (v. 4.5.3, R Core Team, Vienna, Austria) with ggplot2 (v. 4.0.3) and mcr (v. 1.3.1).

## 3. Results

### 3.1. Operational Observations

Given our interest in evaluating the future potential of the TrueSpin centrifuge (Figure 1A) for POC plasma separation of capillary blood collected in Microtainers, we evaluated operational variables and possible failure modes relevant to use in this context.

In total, four TrueSpin centrifuges were used for the entire study. Most Microtainers were spun within 10 min of collection, with a maximum interval prior to spin of 21 min after collection. The TrueSpin begins to spin when the lid is closed and runs until it stops automatically, without operator intervention. Centrifugation time for all 3 Microtainer types ranged from 10 min, 1 s to 10 min, 10 s.

Prior to each use, battery life in the TrueSpin was tested by closing the lid briefly to ensure that a spin was initiated without a “replace battery” error (slow red blinking light). The TrueSpin centrifuges were initially shipped with Alkaline AA batteries, which needed to be changed after one run. After changing the batteries to Energizer Ultimate Lithium AA batteries, each TrueSpin centrifuge supported 8–11 runs before the batteries needed replacement.

There were two instances where the TrueSpin unexpectedly stopped working, though this did not impact any run in progress. In one case, the centrifuge stopped working after only one rotation without a “low battery” error signal; replacement of batteries did not correct the problem, and the centrifuge had to be replaced. In the other case, the centrifuge did not start upon its battery check; this device had recently had its batteries replaced, and the “low battery” error signal was not present. Nonetheless, a second replacement of the batteries caused the centrifuge to run normally again.

Benchtop movement of the TrueSpin during rotation was substantial (frequently “walking” around on the benchtop) but was eliminated by use of a prototype 3D-printed base (Figure 1B) provided by the TrueSpin manufacturer. Because only one base was available, the primary study was performed without the base, and a dedicated experiment was performed to compare the quality of plasma generated with/without the base.

Due to the rapid Microtainer filling process, it was not uncommon to have a small amount of blood on the outside of the Microtainer that needed to be wiped off prior to spin. After centrifugation, a thin ring of blood spatter was observed on the underside of the clear lid of all 4 centrifuges.

### 3.2. Separation Efficacy and Plasma Removal

All TrueSpin plasma separation attempts were visually successful (Figure 1C,D), with obvious separation of plasma from blood components and no visual evidence of hemolysis, except for the very first Microtainer sample collected (an EDTA Microtainer sample which was overfilled to approximately 600 uL) which did not separate. After a second spin of this sample, the plasma was noted to be completely hemolyzed, and it was hypothesized that the original tube may have coagulated either due to overfilling or inadequate inversions. An attempt was made to reproduce this observation by intentionally overfilling one of each type of Microtainer, but plasma separation for those overfilled Microtainers was successful, with no evidence of hemolysis. Nonetheless, after this initial observation, for any Microtainer with blood volume visibly above the maximum fill line (which occurred in 5 out of 38 Microtainer collections), after initial inversion, blood was removed from the Microtainer with a pipettor to reach the maximum fill line. For the LiHep/Gel Microtainer, all TrueSpin centrifugation attempts yielded obvious movement of the cells through the gel.

Plasma was removed from Microtainers with a calibrated pipette. In only one instance, red blood cells were aspirated during plasma removal from an EDTA Microtainer, making the plasma unusable for amino acid analysis. Removal of plasma from the LiHep/Gel Microtainer was noted to be much easier than from the Microtainers without a gel barrier.

### 3.3. Plasma Quality Assessment

Hemolysis indices of plasma generated from all three Microtainers were as follows: EDTA Microtainer, ≤10; LiHep Microtainer, <5; LiHep/Gel Microtainer, ≤6. These H-index values were similar to those of Monovette plasma (EDTA Monovette, 6–24; LiHep/Gel Monovette, ≤10).

Combined concentrations of 24 amino acids measured by LC-UV in plasma generated by TrueSpin centrifugation of each respective Microtainer were correlated with those in paired plasma generated by conventional centrifugation of Monovette samples (Figure 2A–C). Pearson’s correlation coefficients (r) for all sample pairings were >0.99. Passing-Bablok regression analysis yielded a slope of 0.99 (95% CI: 0.98–0.99) and an intercept of 2.32 (95% CI: 1.03–3.61) for the LiHep/Gel Monovette versus LiHep/Gel Microtainer comparison (Figure 2A). The corresponding slope and intercept for the LiHep/Gel Monovette versus LiHep Microtainer comparison (Figure 2B) were 0.99 (95% CI: 0.98–1.00) and 2.18 (95% CI: 0.75–3.61), respectively. For the EDTA Monovette versus EDTA Microtainer comparison (Figure 2C), the slope was 1.02 (95% CI: 1.01–1.03) and the intercept was −0.50 (95% CI: −2.24–1.24). Bland–Altman analysis further demonstrated close agreement between paired plasma specimens, with mean combined amino acid concentration differences of −0.64 µmol/L (LoA: −16.26–14.99 µmol/L) for the LiHep/Gel Monovette versus LiHep/Gel Microtainer comparison (Supplemental Figure S1A), −0.82 µmol/L (LoA: −17.88–16.24 µmol/L) for the LiHep/Gel Monovette versus LiHep Microtainer comparison (Supplemental Figure S1B), and −1.58 µmol/L (LoA: −19.26–16.09 µmol/L) for the EDTA Monovette versus EDTA Microtainer comparison (Supplemental Figure S1C). EDTA Monovette vs. Microtainer comparisons were performed on 7 paired plasma samples (rather than 10) due to three Microtainer sample exclusions: one for complete hemolysis likely due to substantial overfilling (described above), one for red blood cell aspiration during plasma removal (described above), and one for an analytical error that occurred during one of the amino acid analyses.

Plasma generated from TrueSpin centrifugation performed with vs. without the prototype base on duplicate Microtainer specimens collected in series (*n* = 4 EDTA Microtainer pairs, 1 LiHep Microtainer pair, and 1 LiHep/Gel Microtainer pair) had H-indices ≤ 7 and paired samples demonstrated excellent correlation (Pearson’s r = 0.98, 1.0, and 1.0, respectively). Passing-Bablok regression analysis yielded slopes of 0.87 (95% CI: 0.84–0.91), 1.00 (95% CI: 0.99–1.02), and 0.99 (95% CI: 0.98–0.99) for the EDTA, LiHep, and LiHep/Gel microtainer plasma pairs, respectively. These results suggest that the use of the base did not impact the quality of plasma produced by the TrueSpin.

Plasma generated from Microtainers that were intentionally overfilled (*n* = 1 for each of EDTA, LiHep, and LiHep/Gel) had obvious visual separation and H-indices < 5. This indicates that overfilling (while off-label for the Microtainer) did not impact the TrueSpin separation of plasma.

To explore the possibility of a low-cost, POC pathway for capillary blood collection and plasma separation, in *n* = 3 individuals, the Labcorp Comfort Draw system [6,7] was used to collect capillary blood from each arm (one Comfort Draw device per arm) into either a LiHep/Gel or EDTA Microtainer, which was then centrifuged using the TrueSpin (with the base) and resulting plasma collected. Subsequently, blood was collected by routine venipuncture into LiHep/Gel and EDTA Monovettes, and plasma was separated using conventional centrifugation. LiHep/Gel Microtainer fill times using the Comfort Draw device were 1 min 18 s, 1 min 24 s, and 2 min 39 s; EDTA Microtainer fill times were 1 min 37 s, 2 min 08 s, and 4 min 00 s. Extensions of 1–2 min of warming time over the 3 min suggested in the IFU did not consistently reduce filling time. All three volunteers said the lancet and Comfort Draw device were painless and preferred it to venipuncture. LiHep/Gel and EDTA plasma samples from Comfort Draw collection/TrueSpin separation had H-indices ≤5 and ≤13, respectively. These were relatively lower than the H-index values from samples from venipuncture collection/conventional centrifugation (LiHep/Gel, ≤15; EDTA, ≤24). Figure 3A,B and Figure S2A,B compare amino acid concentrations in Microtainer and Monovette plasma samples paired by anticoagulant. Combined amino acid concentrations were highly correlated between the Comfort Draw/Microtainer/TrueSpin and venipuncture/Monovette/conventional centrifugation specimens, with Pearson’s r values of 0.995 for LiHep/Gel and 0.993 for EDTA, respectively. Passing–Bablok regression demonstrated similar relationships between the two blood collection and processing methods. For LiHep/Gel specimens, the slope was 0.94 (95% CI: 0.92–0.97) and the intercept was 13.53 (95% CI: 9.20–17.87) (Figure 3A), while for EDTA specimens, the corresponding slope and intercept were 0.94 (95% CI: 0.91–0.96) and 15.51 (95% CI: 10.43–20.60), respectively (Figure 3B). Bland–Altman analysis (Monovette/conventional—Microtainer/TrueSpin centrifugation) demonstrated a negative bias for both paired comparisons, with mean concentration differences of −6.63 µmol/L (LoA: −38.89–25.63 µmol/L) for LiHep/Gel and −7.87 µmol/L (LoA: −45.16–29.42 µmol/L) for EDTA (Supplemental Figures S2A and S2B, respectively). Although correlation was high, the observed negative bias and relatively wide LoA indicate individual amino acid differences between capillary and venous samples. With the exception of Gln and Hyp, all measured amino acids were higher in capillary than in venous blood. However, our study was small and cannot determine significant differences for individual amino acids. Saito et al. used liquid chromatography–tandem mass spectrometry (LC–MS/MS) to quantify plasma amino acids in paired capillary and venous blood and reported 13 amino acids with statistically significant differences between the sample types, with Asp, Glu, Orn, and Ser higher in capillary blood samples [8]. These findings suggest that the plasma AA biases observed in our paired LiHep/Gel and EDTA comparisons are attributable to inherent differences between capillary and venous blood rather than to the blood processing methods themselves.

## 4. Discussion

For many tests, including those designed for blood analytes relevant to POC testing, plasma or serum are more favorable matrices than whole blood. Hemoglobin may be inhibitory to some assays, particularly those which require highly sensitive or quantitative detection, and cellular components can sometimes confound analyte measurements. In vitro hemolysis can occur during the preanalytical phase of clinical testing and is identified in isolated serum/plasma by the red discoloration resulting from the presence of hemoglobin. Because the clotting required to generate a serum sample can require extended time, plasma is a more feasible sample type for the rapid test turnaround times expected at POC. The current lack of simple solutions for separation of capillary whole blood into liquid plasma that can be used for POC testing remains a barrier to development and deployment of POC blood tests. Notably, none of the current large-volume capillary blood production devices can currently convert blood to liquid plasma, and existing devices for liquid plasma separation are small-volume and expensive [1].

In central laboratories, anticoagulated samples of blood collected by venipuncture into evacuated collection tubes are routinely centrifuged to separate plasma, and the use of collection tubes with gel barriers directly facilitates removal of plasma without contamination by clot debris. Capillary collection containers used for POC collection of large-volume capillary samples can also be centrifuged, but there is currently no precedent for use of a centrifuge for separation of liquid plasma at the POC for waived testing. Concerns about POC centrifugation include the fact that multiple operator steps are currently required to balance and run a centrifuge, increasing both risk and complexity [1]. Development of a Microtainer centrifugation strategy proven to be both simple and safe for the operator would open the door to POC testing of plasma, transforming the field.

The TrueSpin centrifuge has been demonstrated for production of high-quality serum in both POC and home settings [3,4], but has not previously been evaluated for plasma production. Our study, utilizing H-index and an amino acid (*n* = 24) panel to assess plasma quality, indicates that the quality of plasma generated by TrueSpin centrifugation of Microtainer whole blood samples is comparable to the quality of plasma generated by conventional centrifugation of Monovette samples. This was true for all three types of Microtainers, despite differences in required collection volume range (minimum volume ranging from 200–400 μL) and anticoagulant (heparin or EDTA). Given the strength of all correlations observed for amino acids in Microtainer versus Monovette plasma, we were able to draw preliminary conclusions about plasma quality even with a small study.

While the TrueSpin was quite reliable and easy to use, our observations suggest that some improvements are necessary. First, benchtop movement during rotation was significant in the absence of a stabilizing base but was completely eliminated with the addition of a prototype base, with no impact on plasma quality. The base would need to be part of the final product. Second, the two brief run failures upon lid closure (described in Results) may have been related to a “two-tap” procedure noted in the instructions for turning off the TrueSpin after use; these instructions will need to be clarified, and an alternative strategy for centrifuge storage between uses may need to be developed. In terms of user safety, we did notice a faint blood spatter ring on the underside of the lid post-centrifugation, likely due to a small amount of residual blood on the outside of the Microtainer related to our blood draw method (dripping). This would be an issue with any centrifuge and was likely just noticeable due to the clear TrueSpin lid. However, this observation highlights the need for high-quality, no-leak capillary blood collection containers to support POC centrifugation.

Unrelated to the TrueSpin itself, but critical to future use of capillary collection container centrifugation for POC plasma separation, is the importance of having a gel barrier to facilitate plasma removal after centrifugation. While it was simple to remove plasma when a gel barrier was present (and this removal could easily be done with a non-calibrated transfer pipette), it was difficult to remove plasma from Microtainers without the gel barrier, with risk of either aspirating blood cells from the bottom of the tube or wasting plasma by collecting less in order to avoid this aspiration. To facilitate use of Microtainers (or similar capillary collection containers) for POC molecular testing, we strongly suggest development of an EDTA Microtainer with a gel barrier, similar to the LiHep/Gel Microtainer. There is already precedent for plasma preparation tubes that combine EDTA coating with a gel barrier.

In order to focus our main evaluation on the quality of plasma generated by TrueSpin centrifugation versus conventional centrifugation, we used venipuncture blood as our matrix for both Microtainer and Monovette separation in order to eliminate the additional variable of capillary blood collection. Because the ultimate goal would be to use the TrueSpin for capillary blood produced via fingerstick or arm-based lancet tools and collected into the same Microtainers, we did a small proof-of-principle experiment using the Comfort Draw device to collect capillary blood into the Microtainers prior to TrueSpin. Based on hemolysis and amino acid measurements, we found that the quality of plasma generated using this pathway was again comparable to the quality of plasma generated by conventional venipuncture/centrifugation, providing support for a future pathway to generate large-volume capillary plasma samples for testing at POC. Moreover, we found the Comfort Draw device to be painless, rugged, and easy to use, providing motivation for further study of this system.

Our study had some limitations. First, in some cases we removed excess volume from the Microtainer before spinning it so that we were operating within the cleared volume specifications of the Microtainer. We performed limited overfilling experiments that indicated that plasma separation by centrifugation was successful despite overfilling, but did observe one tube for which overfilling led to complete hemolysis. While these issues are inherent to the Microtainer and not specific to the TrueSpin, overfilling is relevant to the overall feasibility of any POC strategy utilizing Microtainers (or similar containers), and dedicated overfilling experiments would need to be done to fully evaluate this possible failure mode. Second, while we removed plasma with a calibrated pipette in this study, POC use would require removal with a non-calibrated tool like a transfer pipette. As above, we believe that the POC use case requires development of an EDTA Microtainer with a gel barrier. Third, while we selected hemolysis and amino acid measurements as our indicators of plasma quality, additional studies utilizing alternative quality measures and analytes would help support the generalizability of our findings.

## 5. Conclusions

In conclusion, our results indicate that the TrueSpin centrifuge is capable of generating high-quality plasma from whole blood collected into a Microtainer and thus support the possibility of future use of the TrueSpin centrifuge for POC plasma separation and testing. While preliminary, our promising results for the combination of the Comfort Draw and TrueSpin suggest a possible future pathway for low-cost, comfortable, and simple collection and separation of capillary blood to facilitate POC testing of plasma. Future studies should include testing for other analytes as measures of plasma quality and additional evaluation of the Comfort Draw (including in pediatric populations) to expand on these findings.

## Figures and Tables

**Figure 1:**
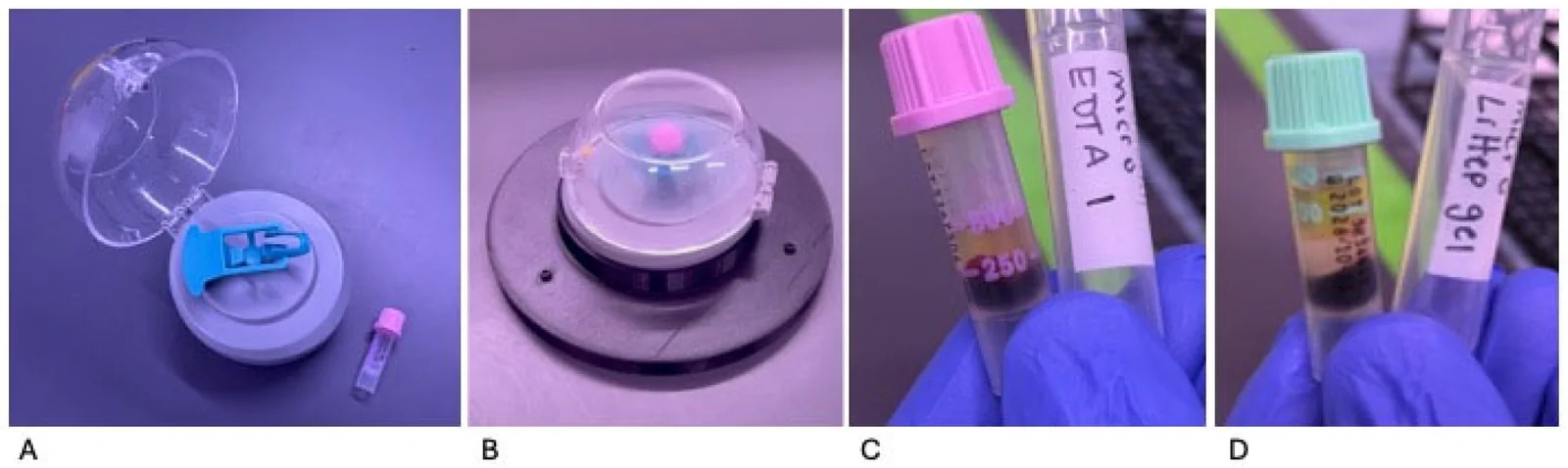
(**A**) The TrueSpin centrifuge and an EDTA Microtainer. (**B**), The TrueSpin centrifuge in operation within a prototype base. (**C**), Representative image of EDTA Microtainer following centrifugation, showing separation of plasma. (**D**), Representative image of LiHep/Gel Microtainer following centrifugation, showing separation of plasma and movement of cellular components through gel to bottom of tube.

**Figure 2:**
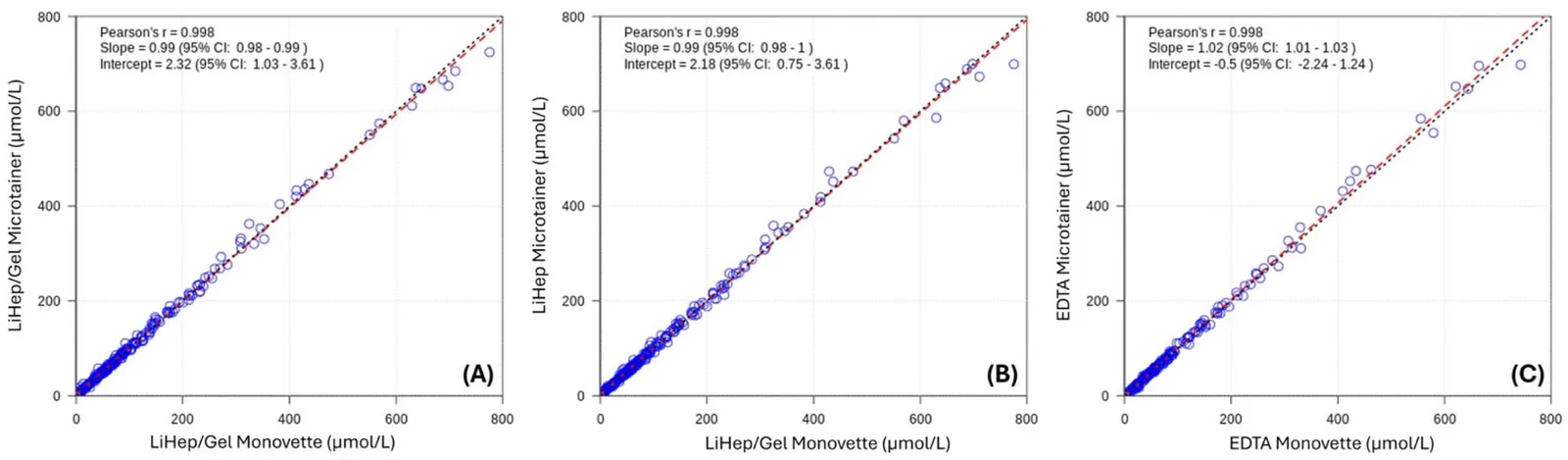
Passing-Bablok regression plots comparing amino acid concentrations in paired plasma samples generated from venous blood processed using Monovette tubes with conventional centrifugation versus Microtainer tubes with TrueSpin centrifugation. Each Passing-Bablok plot shows the line of identity (black dotted line), regression line (red dashed line), and 95% confidence intervals for the slope and intercept. Each plot includes the concentrations of all plasma amino acids (*n* = 24) measured in each set of paired samples. (**A**), LiHep/Gel Monovette vs. LiHep/Gel Microtainer (*n* = 10 sample pairs); (**B**), LiHep/Gel Monovette vs. LiHep Microtainer (*n* = 10 sample pairs); (**C**), EDTA Monovette vs. EDTA Microtainer (*n* = 7 sample pairs). EDTA, Ethylenediaminetetraacetic acid; LiHep, Lithium heparin.

**Figure 3:**
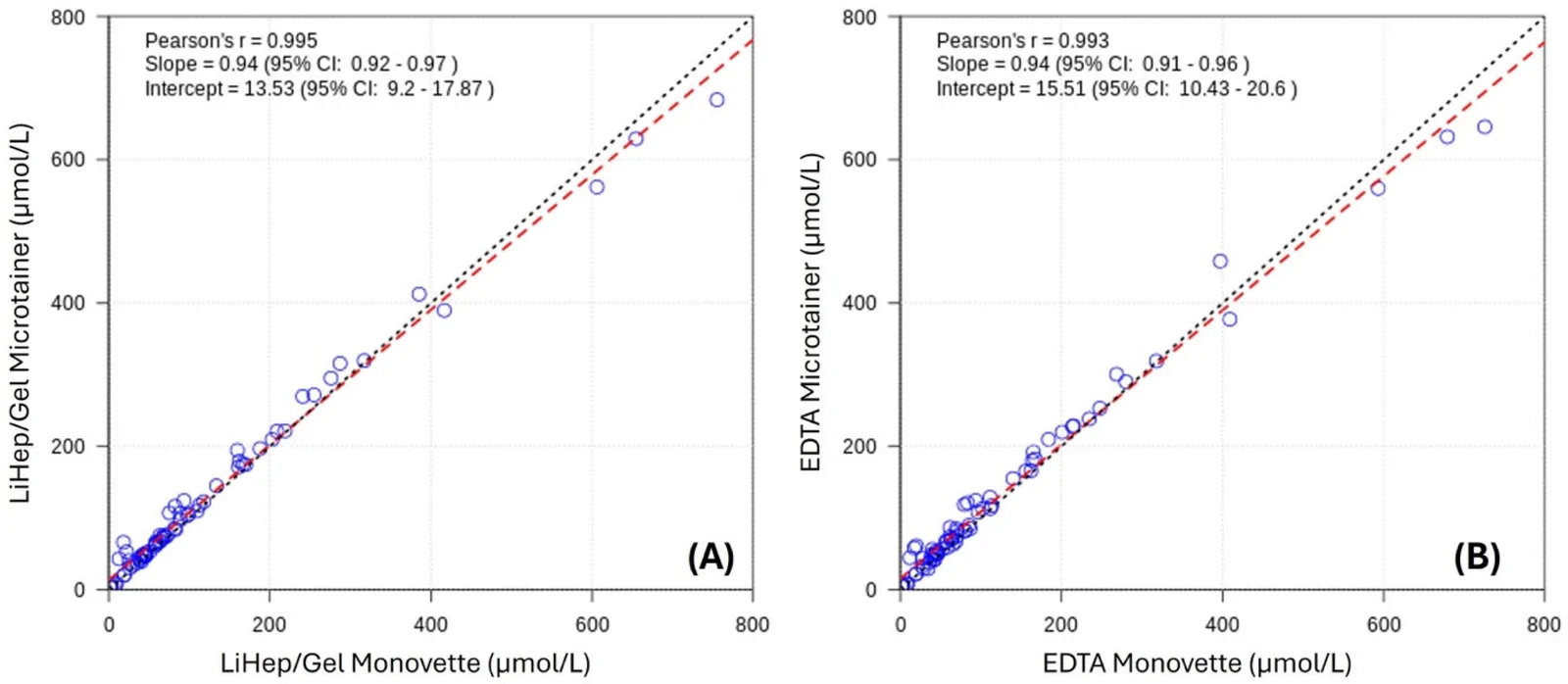
Passing-Bablok regression plots comparing amino acid concentrations in paired plasma samples obtained using two blood collection and processing methods. Venous blood was collected by venipuncture into Monovette tubes and processed by conventional centrifugation. Capillary blood was collected using the Comfort Draw arm-based device into Microtainer tubes and processed by TrueSpin centrifugation. Each Passing-Bablok plot shows the line of identity (black dotted line), regression line (red dashed line), and 95% confidence intervals for the slope and intercept. Each plot includes the concentrations of all plasma amino acids (*n* = 24) measured in each set of paired samples. (**A**), LiHep/Gel Monovette vs. LiHep/Gel Microtainer (*n* = 3 sample pairs); (**B**) EDTA Monovette vs. EDTA Microtainer (*n* = 3 sample pairs). EDTA, Ethylenediaminetetraacetic acid; LiHep, Lithium heparin.

## Data Availability

The original contributions presented in this study are included in the article/Supplementary Materials. Further inquiries can be directed to the corresponding authors.

## Supplementary Materials

The following supporting information can be downloaded at: https://www.mdpi.com/article/10.3390/diagnostics16193088/s1, Supplementary Figure S1. Bland–Altman plots comparing amino acid concentrations in paired plasma samples generated from venous blood processed using Monovette tubes with conventional centrifugation versus Microtainer tubes with TrueSpin centrifugation. Each Bland-Altman plot shows the mean concentration difference and limits of agreement for all plasma amino acids (*n* = 24) measured in each set of paired samples. (A), LiHep/Gel Monovette vs. LiHep/Gel Microtainer (*n* = 10 sample pairs); (B), LiHep/Gel Monovette vs. LiHep Microtainer (*n* = 10 sample pairs); (C), EDTA Monovette vs. EDTA Microtainer (*n* = 7 sample pairs). LOA, limit of agreement; EDTA, Ethylenediaminetetraacetic acid; LiHep, Lithium heparin. Supplementary Figure S2. Bland–Altman plots comparing amino acid concentrations in paired plasma samples obtained using two blood collection and processing methods. Venous blood was collected by venipuncture into Monovette tubes and processed by conventional centrifugation. Capillary blood was collected using the Comfort Draw arm-based device into Microtainer tubes and processed by TrueSpin centrifugation. Each Bland-Altman plot shows the mean concentration difference and limits of agreement for all measured plasma amino acids (*n* = 24). (A), LiHep/Gel Monovette vs. LiHep/Gel Microtainer (*n* = 3 sample pairs); (B), EDTA Monovette vs. EDTA Microtainer (*n* = 3 sample pairs). LOA, limit of agreement; EDTA, Ethylenediaminetetraacetic acid; LiHep, Lithium heparin.

## References

[1] Damhorst G.L., Rao A., Pollock N.R. (2026). More than a drop of blood: The paradigm shift of a large-volume capillary sample for point-of-care clinical diagnostics. J. Clin. Microbiol..

[2] Cepheid Xpert HCV Instructions for Use. https://www.cepheid.com/content/dam/www-cepheid-com/documents/documents/xpert-hcv/303-3318-Rev-A-Xpert-HCV-IFU.pdf.

[3] Iacovetti G., Collier B., Setzer S., Peevler M., Ragar J., Hong K., Pan T., Brandon W., Chappell M., Grant R.P. (2023). Evaluation of a Compact, Portable Centrifuge for Separating Microvolume Blood Samples at the Point of Collection. J. Appl. Lab. Med..

[4] Iacovetti G., Tokunaga N., Peevler M., Ragar J., Sommer G.J., Schaff U.Y. (2024). A-357 evaluation of a home kit for lay user collection, centrifugation, and refrigeration of capillary blood specimens. Clin. Chem..

[5] Peake R.W., Law T., Hoover P.N., Gaewsky L., Shkreta A., Kellogg M.D. (2013). Improved separation and analysis of plasma amino acids by modification of the MassTrak™ AAA Solution Ultraperformance® liquid chromatography method. Clin. Chim. Acta.

[6] Collier B.B., Brandon W.C., Chappell M.R., Iacovetti G., Tokunaga N., Schaff U.Y., Sommer G.J., Grant R.P. (2025). Comparing capillary blood collection technologies: Assessing patient experience, device performance, & clinical accuracy. Bioanalysis.

[7] Schaff U.Y., Collier B.B., Iacovetti G., Peevler M., Ragar J., Tokunaga N., Brandon W.C., Chappell M.R., Grant R.P., Sommer G.J. (2025). Evaluation of a Simplified Upper Arm Device for Vacuum-Assisted Collection of Capillary Blood Specimens. Diagnostics.

[8] Saito K., Ueno S., Nakayama A., Nitta S.I., Arai K., Hasunuma T., Saito Y. (2019). Overall Similarities and a Possible Factor Affecting Plasma Metabolome Profiles Between Venous and Capillary Blood Samples From 20 Healthy Human Males. J. Pharm. Sci..

