# Supplementary material for: Toward the Use of Centrifugation for Point-of-Care Plasma Separation: Evaluation of a Compact Battery-Powered Centrifuge
Source: Diagnostics (Basel). 2026 Sep 24;16(19):3088. doi: 10.3390/diagnostics16193088 (PMC13664047; doi:10.3390/diagnostics16193088)
Supplement: Supplementary file 1 [file diagnostics-16-03088-s001.zip › diagnostics-4500562-supplementary.pdf]

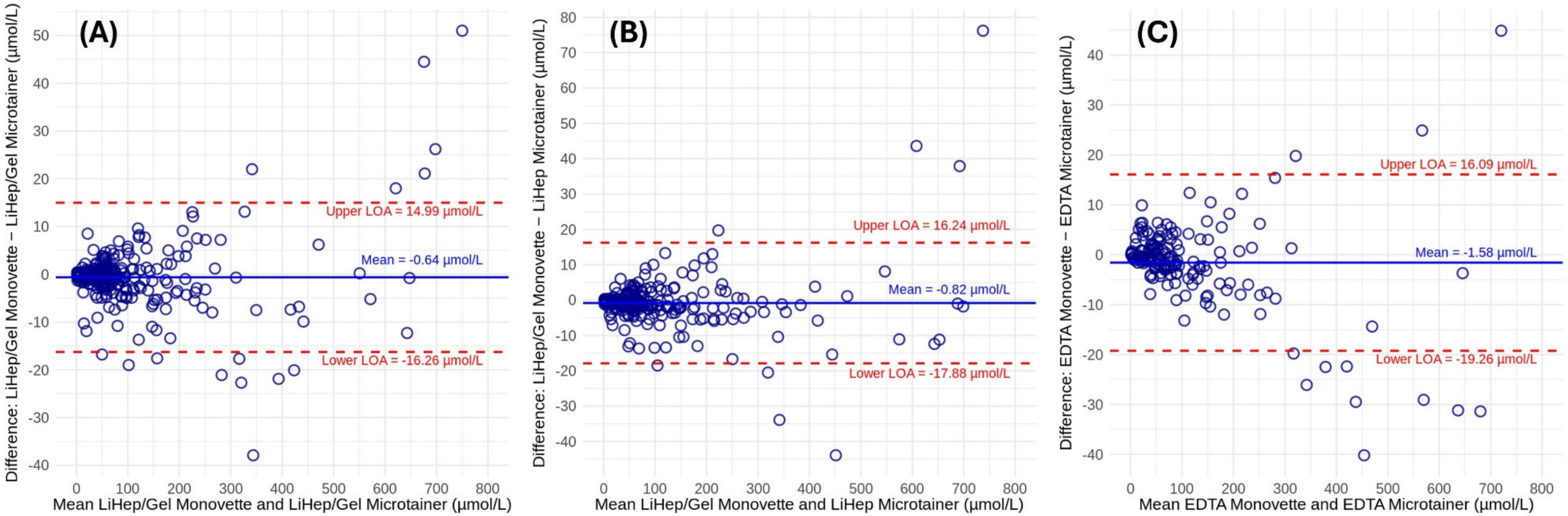

Supplementary Figure S1. Bland–Altman plots comparing amino acid concentrations in paired plasma samples generated from venous blood processed using Monovette tubes with conventional centrifugation versus Microtainer tubes with TrueSpin centrifugation. Each Bland-Altman plot shows the mean concentration difference and limits of agreement for all plasma amino acids (*n* = 24) measured in each set of paired samples. (A), LiHep/Gel Monovette vs. LiHep/Gel Microtainer (*n* = 10 sample pairs); (B), LiHep/Gel Monovette vs. LiHep Microtainer (*n* = 10 sample pairs); (C), EDTA Monovette vs. EDTA Microtainer (*n* = 7 sample pairs). LOA, limit of agreement; EDTA, Ethylenediaminetetraacetic acid; LiHep, Lithium heparin.

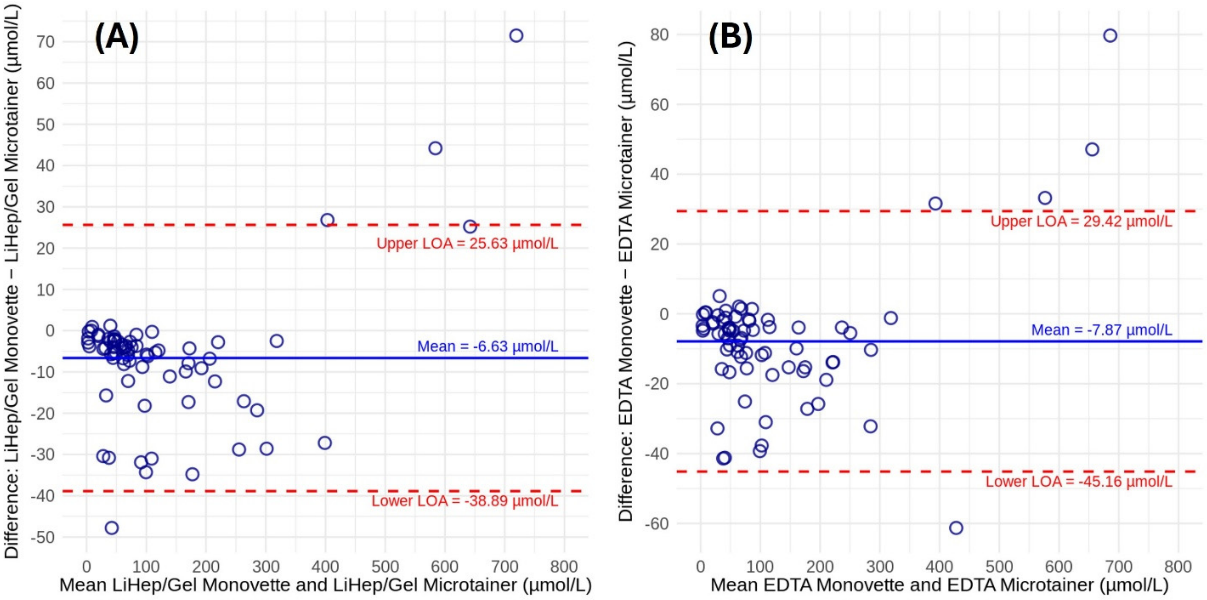

Supplementary Figure S2. Bland–Altman plots comparing amino acid concentrations in paired plasma samples obtained using two blood collection and processing methods. Venous blood was collected by venipuncture into Monovette tubes and processed by conventional centrifugation. Capillary blood was collected using the Comfort Draw arm-based device into Microtainer tubes and processed by TrueSpin centrifugation. Each Bland-Altman plot shows the mean concentration difference and limits of agreement for all measured plasma amino acids (*n* = 24). (A), LiHep/Gel Monovette vs. LiHep/Gel Microtainer (*n* = 3 sample pairs); (B), EDTA Monovette vs EDTA Microtainer (*n* = 3 sample pairs). LOA, limit of agreement; EDTA, Ethylenediaminetetraacetic acid; LiHep, Lithium heparin.
